# Repeatability and reproducibility of a new spectral-domain optical coherence tomography biometer and agreement with swept-source optical coherence tomography based biometer

**DOI:** 10.1186/s40662-024-00422-0

**Published:** 2025-02-01

**Authors:** Xin Li, Chak Seng Lei, Rui Ning, Luze Liu, Aodong Chen, Xinning Yang, Giacomo Savini, Domenico Schiano-Lomoriello, Xingtao Zhou, Jinhai Huang

**Affiliations:** 1 Eye Institute and Department of Ophthalmology and Vision Science, Eye & ENT Hospital, Fudan University, Shanghai, 200031 China; 2 NHC Key Laboratory of Myopia and Related Eye Diseases, Key Laboratory of Myopia and Related Eye Diseases, Chinese Academy of Medical Sciences, Shanghai, 200031 China; 3 Shanghai Research Center of Ophthalmology and Optometry, Shanghai, China; 4 Eye Hospital and School of Ophthalmology and Optometry, Wenzhou Medical University, Wenzhou, Zhejiang China; 5 School of Health Science and Engineering, University of Shanghai for Science and Technology, Shanghai, China; 6 G.B. Bietti Foundation I.R.C.C.S, Rome, Italy

**Keywords:** Ocular biometry, Repeatability, Reproducibility, Agreement, Optical coherence tomography

## Abstract

**Background:**

To assess the repeatability and reproducibility of the Colombo IOL biometer (Moptim, China), which utilizes spectral-domain optical coherence tomography (SD-OCT), in measuring ocular parameters of normal subjects and to compare its agreement with the swept-source optical coherence tomography (SS-OCT)-based IOLMaster 700 biometer (Carl Zeiss Meditec AG, Germany).

**Methods:**

This prospective study included 91 eyes from 91 normal subjects. The evaluated parameters were axial length (AL), central corneal thickness (CCT), aqueous depth (AQD), anterior chamber depth (ACD), lens thickness (LT), flattest and steepest meridian keratometry (Kf and Ks), mean keratometry (Km), astigmatism (AST) magnitude, white-to-white (WTW) distance, and pupil diameter (PD). The within-subject standard deviation (S_w_), test–retest repeatability (TRT), coefficient of variation (CoV), and intraclass correlation coefficient (ICC) were calculated to determine the repeatability and reproducibility. Paired t-tests and Bland–Altman plots with 95% limits of agreement (LoA) were employed to assess the agreement.

**Results:**

With respect to intraobserver repeatability, the S_w_ and TRT values of all evaluated parameters were low. Except J_45_ and PD, the ICCs were all higher than 0.928. The reproducibility S_w_ and TRT values of Colombo IOL were also low, and ICCs were not lower than 0.900. Comparing Colombo IOL and IOLMaster 700, the 95% LoA of AL, CCT, AQD, ACD, LT, Kf, Ks, Km, AST, J_0_, J_45_, WTW and PD ranged from − 0.08 to 0.03 mm, − 21.58 to 5.09 μm, 0.01 to 0.15 mm, − 0.01 to 0.14 mm, − 0.05 to 0.10 mm, − 0.14 to 0.59 D, − 0.31 to 0.40 D, − 0.13 to 0.40 D, − 0.68 to 0.32 D, − 0.09 to 0.34 D, − 0.07 to 0.25 D, 0.11 to 1.47 mm, and − 0.97 to 2.31 mm, respectively.

**Conclusion:**

The new SD-OCT-based Colombo IOL biometer demonstrates excellent repeatability and reproducibility. Moreover, it generally agrees well with the SS-OCT-based IOLMaster 700, except for the WTW and PD measurements.

## Background

Accurate measurement of eye parameters is crucial for the diagnosis and management of ocular disorders, including procedures such as cataract surgery, refractive surgery, and sizing of phakic implantable lenses. These measurements are vital for preoperative evaluations and designing surgical plans in refractive surgery. Consequently, biometric technologies are continuously evolving and are increasingly employed in clinical settings [1–8]. Optical coherence tomography (OCT) is an advanced ocular imaging technology offering high-resolution visualizations via non-invasive means, thereby serving as an indispensable tool for ophthalmic diagnostics and research [9].

Time-domain OCT (TD-OCT) is one type of OCT that captures images by allowing the light reflected from intraocular tissues to overlap and interfere with reference system light, achieved by moving the reference arm. Fourier-domain OCT (FD-OCT) represents another category, which comprises spectral-domain OCT (SD-OCT) and swept-source OCT (SS-OCT) [10]. Biometers based on OCT technology have demonstrated high precision and accurate measurements for IOL power calculation [11, 12]. The IOLMaster 700 (Carl Zeiss Meditec AG, Jena, Germany) was the first SS-OCT-based optical biometer. The consistency in its repeatability and its correlation with other instruments have been validated [13, 14]. The Colombo IOL (Moptim, China) is a new optical biometer that utilizes SD-OCT with an 850-nm scanning light source to obtain comprehensive ocular biological parameters through OCT in a single acquisition.

New instruments must be thoroughly evaluated before clinical use to ensure their performance and precision, as well as to assess their potential as substitutes for other instruments. The study aims to evaluate the repeatability and reproducibility of the new SD-OCT-based biometer and its agreement with the SS-OCT-based biometer in normal subjects.

## Methods

### Subjects

The study received approval from the Institutional Ethics Committee at the Eye and ENT Hospital of Fudan University (No. 2021175). It adhered strictly to the principles of the Declaration of Helsinki and underwent thorough ethical review. Participants were provided with comprehensive details about the study's objectives and methods and gave their written consent by signing an informed consent document before participating. All subjects received a full ophthalmic examination that included manifest refraction, slit-lamp microscopy, fundus photography, and non-contact tonometry. The inclusion criteria were—a minimum age of 18 years, the ability to cooperate with all test procedures, stable visual fixation, best-corrected distance visual acuity of 20/20 or better, able to stop wearing soft contact lenses for at least two weeks and hard contact lenses for at least four weeks. Exclusion criteria were—any history of keratitis, cataract, glaucoma, keratoconus, vitreoretinal diseases, previous ocular trauma or surgery, significant corneal opacity, or dry eyes.

### Instruments

**Fig. 1:**
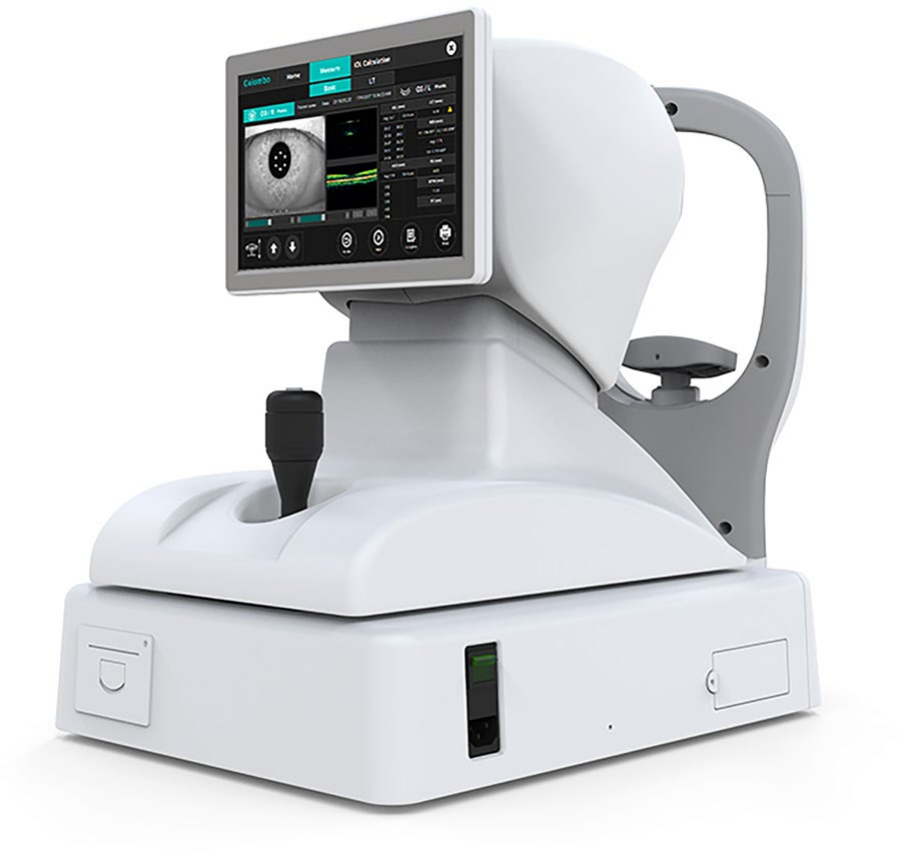
Device diagram of the Colombo IOL

**Fig. 2:**
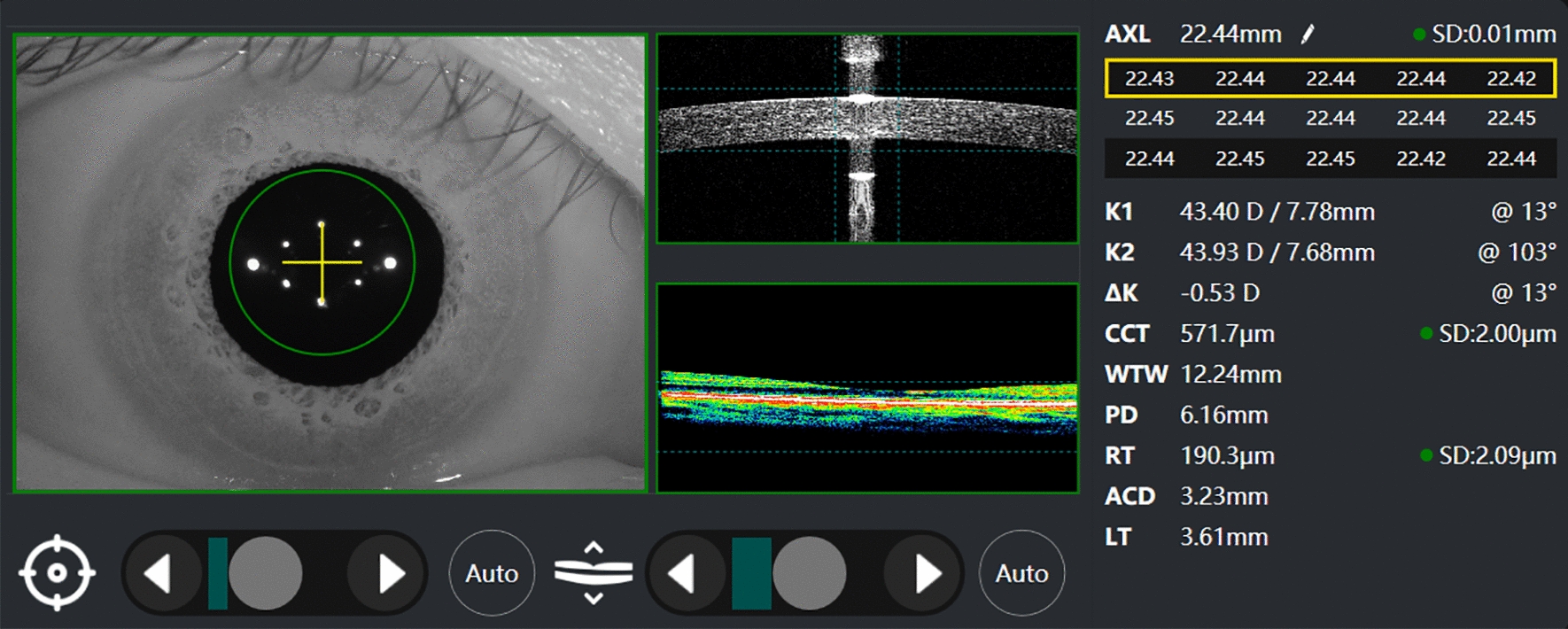
Measurement interface of the Colombo IOL

The new Colombo IOL (Figs. 1, 2) adopts high-resolution SD-OCT technology and uses a scanning light source with a wavelength of 850 nm to provide cross-sectional images with an axial resolution of 5 μm. Axial length (AL), central corneal thickness (CCT), anterior chamber depth (ACD), aqueous depth (AQD), keratometry (K), corneal astigmatism (AST), white-to-white (WTW), and pupil diameter (PD) can be obtained in a single acquisition. AL is measured 15 times in one single scan and the mean value is reported. The K value is determined by analyzing the reflection of six light points, symmetrically arranged in a hexagonal pattern with a 2.3-mm diameter. WTW and PD are measured with a high-resolution camera to detect the corneal and pupil’s boundary. The Colombo IOL can provide real-time imaging of the 3 mm retina during measurement process to aid the examiner in determining the subject’s fixation state with macular confirmation at the point of acquisition.

The IOLMaster 700 employs SS-OCT technology with a 1050-nm wavelength light. It operates at a scanning speed of 2000 A-scans per second and a depth of 44 mm, providing comprehensive images from cornea to retina. The device’s anterior segment scan covers a 6-mm width with a tissue resolution of 22 μm. It acquires three B-scans in each of the six meridians of the cornea, yielding a total of 18 measurements. K value is determined from 18 telecentric measurements taken at three different ring diameters (1.5, 2.5, and 3.5 mm). Additionally, the IOLMaster 700 features a light emitting diode (LED) light source for measuring WTW and PD.

### Measurement procedure

To prevent measurement bias, the order of measurements was randomized using a computer-generated number table. All assessments were taken between 10 a.m. and 5 p.m., ensuring that patients were fully awake for at least two hours beforehand. Measurements were conducted by a trained operator using both devices, a second operator measured the same subjects using the Colombo IOL. The procedure was conducted as follows: in a dimly lit room, the operator adjusted the device's jaw support to align the outer canthus with the marker line, and then prompted the patient to lean their forehead against the support. During measurements, patients were instructed to keep both eyes open, focus on a designated point, and blink completely to ensure an even tear film. After each measurement, patients rested their eyes briefly [15]. Only scans with high image quality were selected for analysis, three consecutive results were required for each device. The mean of three measurements taken by each operator using the Colombo IOL device was used to analyze repeatability and reproducibility. The mean of three measurements from each device, performed by the same operator, was used to compare inter-device differences. In this study, we considered several parameters: AL, CCT, AQD, ACD, LT, flattest meridian K (Kf), steepest meridian K (Ks), mean K (Km), keratometric AST, WTW, and PD. AST was analyzed by J_0_ and J_45_ vectors projections according to the formulas J_0_ = − (AST/2) cos 2θ and J_45_ = − (AST/2) sin 2θ [16].

### Statistical analysis

SPSS software (version 21.0; IBM Corporation, Armonk, NY) and MedCalc Statistical software (version 19.1.3; MedCalc Software Inc., Mariakerke, Belgium) were used for data analysis. The data were normally distributed, permitting the use of parametric tests. To evaluate the repeatability and reproducibility levels of the Colombo IOL, several metrics were employed: within-subject standard deviation (S_w_), test–retest repeatability (TRT), and coefficient of variation (CoV), along with the intraclass correlation coefficient (ICC). S_w_, the square root of the within-group mean square deviation, indicates the extent of variability within the group. TRT, calculated as 2.77 × S_w_, defines the 95% confidence interval for the difference between measurements and depicts the range of results across multiple tests. Lower values of S_w_ and TRT indicate superior repeatability and reproducibility [17]. CoV is the ratio of S_w_ to the mean, determining the relative degree of variation in the data. Smaller CoV values indicate better repeatability and reproducibility. The ICC was calculated using the two-way mixed model and absolute agreement, it quantifies the reliability of measurement results. An ICC value close to 1.00 signifies minimal variability, and thus higher reliability. Typically, an ICC above 0.90 suggests excellent reproducibility, between 0.75 and 0.90 indicates moderate reproducibility, and below 0.75 reflects poor reproducibility [18, 19]. Paired t-tests were used to assess the mean difference (MD) between the two devices in each measured parameter to determine whether they were significantly different. A *P* value of less than 0.05 indicates a statistically significant difference.

To evaluate the agreement between the two instruments, Bland–Altman plots and 95% limits of agreement (95% LoA) were utilized [20]. The 95% LoA was calculated as the MD between the devices ± 1.96 × the standard deviation of these differences. A narrower 95% LoA interval indicates better agreement. On a Bland–Altman plot, the x-axis shows the mean of the measurements from the two devices, while the y-axis shows their differences. The solid line depicts the MD, and the dashed lines above and below this represents the 95% LoA [21].

As stated by McAlinden et al. [22], a sample size of 96 eyes achieves a confidence interval of ± 0.10 for the estimated measurements with three repeated measures. Accordingly, our sample size provides a similar level of precision and is considered adequate for the power of this study.

## Results

A total of 91 eyes of 91 subjects were enrolled in the study. Among them, 51 were male and 40 were female. The average age was 28.3 ± 6.9 years (19–48 years). The average sphere power was − 5.09 ± 2.04 diopters (D) (range: − 12.00 D to + 1.00 D) and the average cylinder power was − 0.93 ± 0.76 D (range: − 3.25 D to 0.00 D). The mean spherical equivalent was − 5.56 ± 2.12 D (range: − 12.63 D to − 1.25 D). Tables 1 and 2 summarize the mean, maximum, and minimum values of ocular parameter measurements measured by the two devices.

**Table 1:** Ocular biological parameters measured by the Colombo IOL

Parameter	Mean ± SD	Min	Max
AL (mm)	25.53 ± 1.27	22.33	29.10
CCT (μm)	529.81 ± 32.01	456.20	613.00
AQD (mm)	3.13 ± 0.30	2.20	4.02
ACD (mm)	3.66 ± 0.30	2.70	4.61
LT (mm)	3.82 ± 0.35	3.04	5.03
Kf (D)	43.11 ± 1.33	40.25	46.83
Ks (D)	44.13 ± 1.50	40.85	48.14
Km (D)	43.62 ± 1.39	40.55	47.48
AST (D)	1.02 ± 0.60	0.12	2.87
J_0_ (D)	− 0.39 ± 0.38	− 1.41	0.60
J_45_ (D)	0.14 ± 0.18	− 0.37	0.72
WTW (mm)	12.67 ± 0.55	11.49	14.27
PD (mm)	5.96 ± 1.20	3.04	8.64

*AL* = axial length; *CCT* = central corneal thickness; *AQD* = aqueous humor depth; *ACD* = anterior chamber depth; *LT* = lens thickness; *Kf* = flattest meridian keratometry; *Ks* = steepest meridian keratometry; *Km* = mean keratometry; *AST* = corneal astigmatism; *J*_*0*_ = astigmatism parameter J_0_; *J*_*45*_ = astigmatism parameter J_45_; *WTW* = white to white; *PD* = pupil diameter; *SD* = standard deviation

**Table 2:** Ocular biological parameters measured by the IOLMaster 700

Parameter	Mean ± SD	Min	Max
AL (mm)	25.56 ± 1.28	22.35	29.17
CCT (μm)	544.01 ± 33.71	469.00	629.33
AQD (mm)	3.05 ± 0.3	2.14	3.94
ACD (mm)	3.59 ± 0.31	2.67	4.55
LT (mm)	3.79 ± 0.35	3.03	5.02
Kf (D)	42.90 ± 1.38	40.04	47.06
Ks (D)	44.09 ± 1.55	40.73	47.88
Km (D)	43.49 ± 1.42	40.39	47.47
AST (D)	1.19 ± 0.73	0.05	3.42
J_0_ (D)	− 0.50 ± 0.43	− 1.71	0.54
J_45_ (D)	0.05 ± 0.20	− 0.53	0.60
WTW (mm)	11.98 ± 0.37	11.10	12.77
PD (mm)	5.27 ± 1.15	2.73	8.43

*AL* = axial length; *CCT* = central corneal thickness; *AQD* = aqueous humor depth; *ACD* = anterior chamber depth; *LT* = lens thickness; *Kf* = flattest meridian keratometry; *Ks* = steepest meridian keratometry; *Km* = mean keratometry; *AST* = corneal astigmatism; *J*_*0*_ = astigmatism parameter J_0_; *J*_*45*_ = astigmatism parameter J_45_; *WTW* = white to white; *PD* = pupil diameter; *SD* = standard deviation

### Repeatability results of Colombo IOL measurements

The measurement repeatability results of AL, CCT, AQD, ACD, LT, Kf, Ks, Km, AST, J_0_, J_45_, WTW, and PD are presented in Table 3. The S_w_ values for all parameters were low. TRT values were also low (AL: 0.02–0.03 mm, CCT: 3.26–4.52 μm, AQD: 0.09–0.10 mm, ACD: 0.09–0.10 mm, LT: 0.10–0.12 mm, Kf: 0.33–0.36 D, Ks: 0.36 D, Km: 0.28–0.30 D, AST: 0.40 D, J_0_: 0.22–0.25 D, J_45_: 0.21–0.22 D, WTW: 0.39–0.41 mm, PD: 0.89–1.05 mm). The CoV values were all low, ranging from 0.03% to 6.35%. Among them, the CoV value of AL parameter was the lowest, with only 0.03%. The ICC values of AL, CCT, AQD, ACD, LT, Kf, Ks, Km, AST, J0 and WTW, were all higher than 0.900. The ICC value of AL was the highest (1.000), and the ICC values of J_45_ and PD were 0.849 and 0.875, respectively. In conclusion, ocular biological parameters measured by Colombo IOL biometer showed excellent intraobserver repeatability, with AL parameter showing the best performance.

**Table 3:** Results of the repeatability analysis of the Colombo IOL measurements in normal subjects

Parameter		Mean ± SD	S_w_	TRT	CoV (%)	ICC (95% CI)
AL (mm)	1st	25.53 ± 1.27	0.01	0.02	0.03	1.000 (1.000–1.000)
	2nd	25.51 ± 1.14	0.01	0.03	0.04	1.000 (1.000–1.000)
CCT (μm)	1st	529.81 ± 32.01	1.63	4.52	0.31	0.997 (0.996–0.998)
	2nd	531.46 ± 29.41	1.18	3.26	0.22	0.998 (0.998–0.999)
AQD (mm)	1st	3.13 ± 0.30	0.04	0.10	1.18	0.985 (0.979–0.989)
	2nd	3.14 ± 0.30	0.03	0.09	1.03	0.989 (0.983–0.993)
ACD (mm)	1st	3.66 ± 0.30	0.04	0.10	1.01	0.985 (0.980–0.989)
	2nd	3.67 ± 0.31	0.03	0.09	0.88	0.989 (0.984–0.993)
LT (mm)	1st	3.82 ± 0.35	0.04	0.10	0.95	0.989 (0.985–0.992)
	2nd	3.81 ± 0.34	0.04	0.12	1.13	0.984 (0.976–0.990)
Kf (D)	1st	43.11 ± 1.33	0.12	0.33	0.27	0.992 (0.989–0.994)
	2nd	43.13 ± 1.37	0.13	0.36	0.30	0.991 (0.987–0.994)
Ks (D)	1st	44.13 ± 1.50	0.13	0.36	0.29	0.993 (0.990–0.995)
	2nd	44.15 ± 1.50	0.13	0.36	0.30	0.992 (0.989–0.995)
Km (D)	1st	43.62 ± 1.39	0.10	0.28	0.23	0.995 (0.993–0.996)
	2nd	43.64 ± 1.41	0.11	0.30	0.25	0.994 (0.991–0.996)
AST (D)	1st	1.02 ± 0.60	0.15	0.40	14.29	0.943 (0.924–0.959)
	2nd	1.02 ± 0.60	0.15	0.40	14.17	0.943 (0.916–0.963)
J_0_ (D)	1st	− 0.39 ± 0.38	0.08	0.22	− 20.58	0.957 (0.942–0.969)
	2nd	− 0.38 ± 0.38	0.09	0.25	− 23.20	0.946 (0.921–0.964)
J_45_ (D)	1st	0.14 ± 0.18	0.08	0.21	53.89	0.849 (0.801–0.888)
	2nd	0.14 ± 0.19	0.08	0.22	56.26	0.852 (0.789–0.900)
WTW (mm)	1st	12.67 ± 0.55	0.14	0.39	1.10	0.937 (0.916–0.954)
	2nd	12.63 ± 0.53	0.15	0.41	1.16	0.928 (0.894–0.952)
PD (mm)	1st	5.96 ± 1.20	0.32	0.89	5.41	0.931 (0.908–0.950)
	2nd	5.95 ± 1.02	0.38	1.05	6.35	0.875 (0.821–0.917)

*AL* = axial length; *CCT* = central corneal thickness; *AQD* = aqueous humor depth; *ACD* = anterior chamber depth; *LT* = lens thickness; *Kf* = flattest meridian keratometry; *Ks* = steepest meridian keratometry; *Km* = mean keratometry; *AST* = corneal astigmatism; *J*_*0*_ = astigmatism parameter J_0_; *J*_*45*_ = astigmatism parameter J_45_; *WTW* = white to white; *PD* = pupil diameter; *SD* = standard deviation; *Sw* = within-subject standard deviation; *TRT* = test–retest repeatability coefficient (2.77 × Sw); *CoV* = coefficient of variation; *ICC* = intraclass correlation coefficient; *CI* = confidence interval

### Reproducibility results of Colombo IOL measurements

The measurement reproducibility results of AL, CCT, AQD, ACD, LT, Kf, Ks, Km, AST, J_0_, J_45_, WTW, and PD are reported in Table 4. The S_w_ values for all parameters were low, and also with low TRT values (AL: 0.01 mm, CCT: 2.49 μm, AQD: 0.06 mm, ACD: 0.06 mm, LT: 0.08 mm, Kf: 0.24 D, Ks: 0.21 D, Km: 0.19 D, AST: 0.24 D, J_0_: 0.13 D, J_45_: 0.10 D, WTW: 0.24 mm, PD: 0.95 mm), indicating excellent reproducibility. The ICC values of all parameters were not lower than 0.964, which further proved the reliability of these parameters. In addition, we observed that the CoV values were all less than 8.43%, indicating high reproducibility.

**Table 4:** Results of reproducibility analysis of the Colombo IOL measurements in normal subjects

Parameter	Mean ± SD	S_w_	TRT	CoV (%)	ICC (95% CI)
AL (mm)	25.51 ± 1.14	0.00	0.01	0.02	1.000 (1.000–1.000)
CCT (μm)	531.41 ± 29.52	0.90	2.49	0.17	0.999 (0.998–0.999)
AQD (mm)	3.14 ± 0.31	0.02	0.06	0.70	0.995 (0.992–0.997)
ACD (mm)	3.67 ± 0.31	0.02	0.06	0.60	0.995 (0.992–0.997)
LT (mm)	3.81 ± 0.34	0.03	0.08	0.72	0.994 (0.989–0.996)
Kf (D)	43.12 ± 1.37	0.09	0.24	0.20	0.996 (0.994–0.998)
Ks (D)	44.14 ± 1.51	0.08	0.21	0.18	0.997 (0.996–0.998)
Km (D)	43.63 ± 1.41	0.07	0.19	0.16	0.998 (0.996–0.999)
AST (D)	1.02 ± 0.6	0.09	0.24	8.43	0.980 (0.967–0.987)
J_0_ (D)	− 0.38 ± 0.37	0.05	0.13	− 11.77	0.985 (0.977–0.991)
J_45_ (D)	0.14 ± 0.2	0.04	0.10	26.70	0.964 (0.942–0.978)
WTW (mm)	12.63 ± 0.54	0.09	0.24	0.70	0.973 (0.957–0.984)
PD (mm)	5.93 ± 1.05	0.34	0.95	5.77	0.900 (0.842–0.937)

*AL* = axial length; *CCT* = central corneal thickness; *AQD* = aqueous humor depth; *ACD* = anterior chamber depth; *LT* = lens thickness; *Kf* = flattest meridian keratometry; *Ks*: steepest meridian keratometry; *Km* = mean keratometry; *AST* = corneal astigmatism; *J*_*0*_ = astigmatism parameters J_0_; *J*_*45*_ = astigmatism parameters J_45_; *WTW* = white to white; *PD* = pupil diameter; *SD* = standard deviation; *Sw* = within-subject standard deviation; *TRT* = test–retest repeatability coefficient (2.77 × Sw); *CoV* = coefficient of variation; *ICC* = intraclass correlation coefficient; *CI* = confidence interval

### Comparisons between the Colombo IOL and IOLMaster 700

**Fig. 3:**
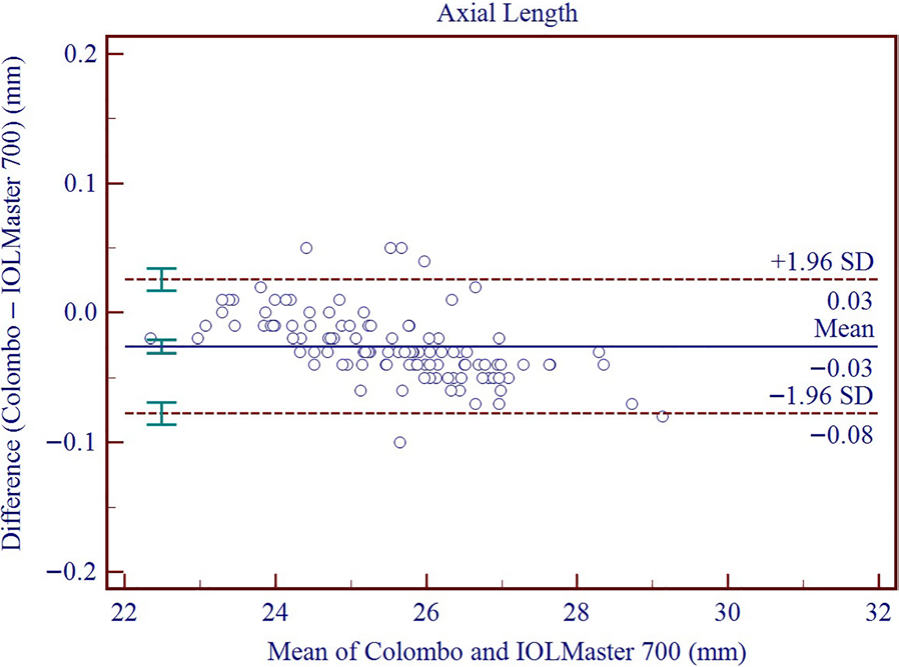
Bland–Altman plots show the agreement between the Colombo IOL and IOLMaster 700 for axial length. The solid line represents the mean difference. The upper and lower dashed lines represent the 95% limits of agreement (LoA). The green error bars represent the 95% confidence interval (CI) for the mean difference and the 95% LoA

**Fig. 4:**
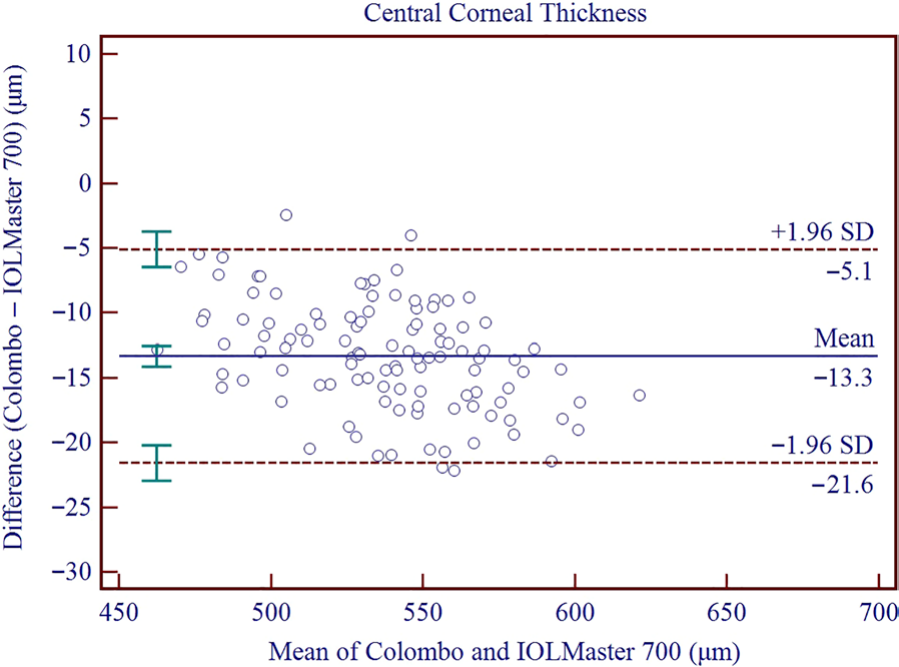
Bland–Altman plots show the agreement between the Colombo IOL and IOLMaster 700 for central corneal thickness. The solid line represents the mean difference. The upper and lower dashed lines represent the 95% limits of agreement (LoA). The green error bars represent the 95% confidence interval (CI) for the mean difference and the 95% LoA

**Fig. 5:**
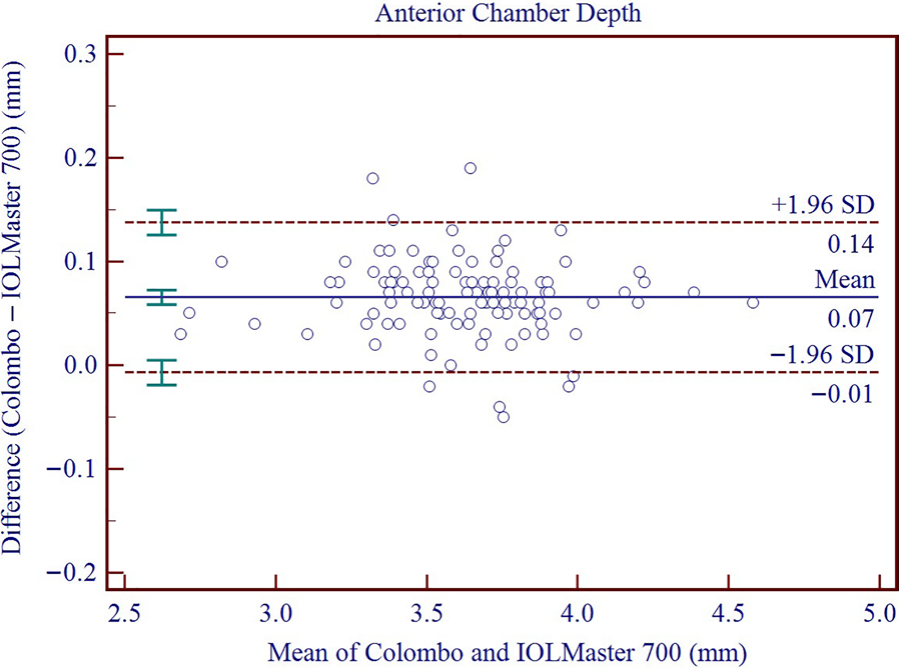
Bland–Altman plots show the agreement between the Colombo IOL and IOLMaster 700 for anterior chamber depth (ACD). The solid line represents the mean difference. The upper and lower dashed lines represent the 95% limits of agreement (LoA). The green error bars represent the 95% confidence interval (CI) for the mean difference and the 95% LoA

**Fig. 6:**
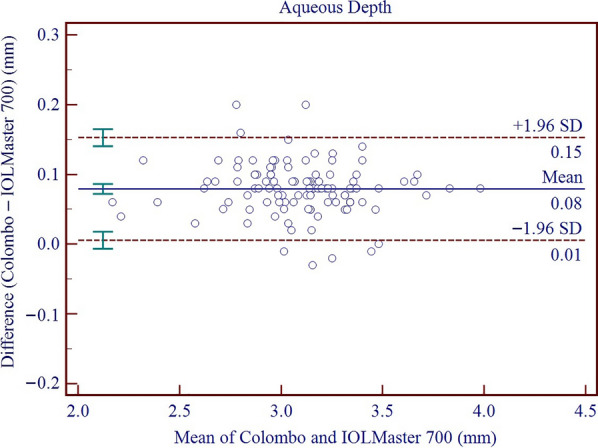
Bland–Altman plots show the agreement between the Colombo IOL and IOLMaster 700 for aqueous depth (AQD). The solid line represents the mean difference. The upper and lower dashed lines represent the 95% limits of agreement (LoA). The green error bars represent the 95% confidence interval (CI) for the mean difference and the 95% LoA

**Fig. 7:**
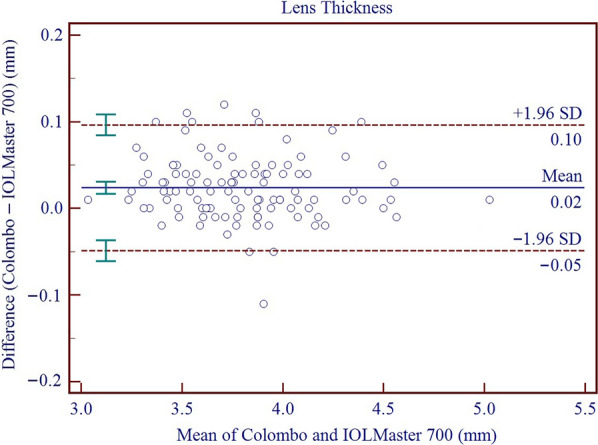
Bland–Altman plots show the agreement between the Colombo IOL and IOLMaster 700 for lens thickness (LT). The solid line represents the mean difference. The upper and lower dashed lines represent the 95% limits of agreement (LoA). The green error bars represent the 95% confidence interval (CI) for the mean difference and the 95% LoA

**Fig. 8:**
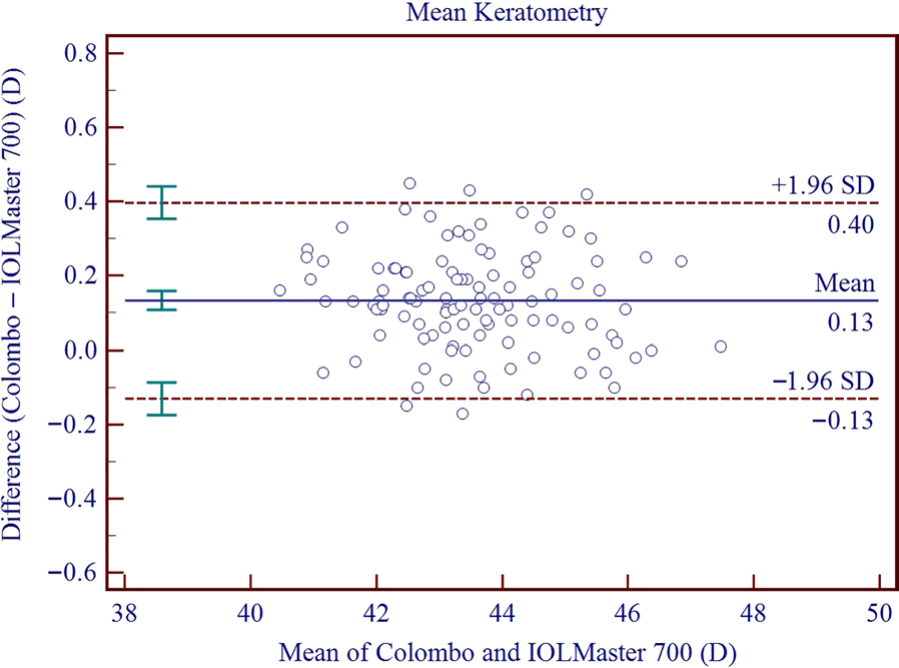
Bland–Altman plots show the agreement between the Colombo IOL and IOLMaster 700 for mean keratometry (Km). The solid line represents the mean difference. The upper and lower dashed lines represent the 95% limits of agreement (LoA). The green error bars represent the 95% confidence interval (CI) for the mean difference and the 95% LoA

**Fig. 9:**
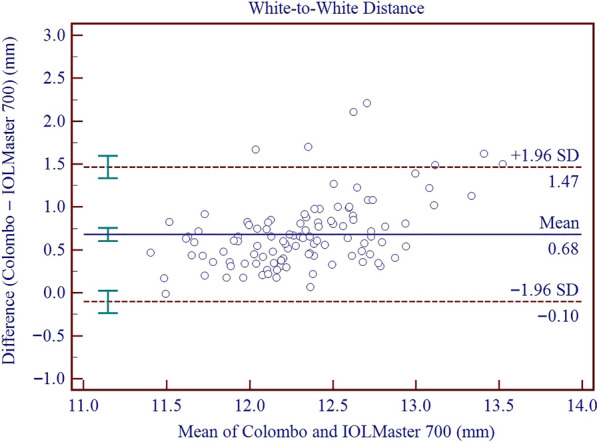
Bland–Altman plots show the agreement between the Colombo IOL and IOLMaster 700 for white-to-white (WTW). The solid line represents the mean difference. The upper and lower dashed lines represent the 95% limits of agreement (LoA). The green error bars represent the 95% confidence interval (CI) for the mean difference and the 95% LoA

Results on the differences and agreement between the two device measurements are presented in Table 5. In this study, the measurements of the Colombo IOL were found to be higher than those of the IOLMaster 700 for AQD, ACD, LT, Kf, Ks, Km, J_0_, J_45_, WTW, and PD, while the measurements of AL and CCT were lower than those of the IOLMaster 700. All these differences were found to be statistically significant. Significant proportional bias was detected for AL (r = − 0.011; *P* < 0.001), CCT (r = − 0.056; *P* < 0.001) and WTW (r = 0.448; *P* < 0.001). The 95% LoA for AL, CCT, AQD, ACD, LT, Kf, Ks, Km, AST, J_0_, J_45_, WTW, and PD ranged from − 0.08 to 0.03 mm, − 21.58 to 5.09 μm, 0.01 to 0.15 mm, − 0.01 to 0.14 mm, − 0.05 to 0.10 mm, − 0.14 to 0.59 D, − 0.31 to 0.40 D, − 0.13 to 0.40 D, − 0.68 to 0.32 D, − 0.09 to 0.34 D, − 0.07 to 0.25 D, 0.11 to 1.47 mm, and − 0.97 to 2.31 mm, respectively. Figures 3, 4, 5, 6, 7, 8 and 9 show Bland–Altman plots of AL, CCT, AQD, ACD, LT, Km, and WTW as obtained by the two instruments.

**Table 5:** Results of agreement analysis between the Colombo IOL and IOLMaster 700 measurements

Parameter	Mean difference ± SD	*P* value	95% LoA
AL (mm)	− 0.03 ± 0.03	0.000	− 0.08 to 0.03
CCT (μm)	− 13.34 ± 4.21	0.000	− 21.58 to − 5.09
AQD (mm)	0.08 ± 0.04	0.000	0.01 to 0.15
ACD (mm)	0.07 ± 0.04	0.000	− 0.01 to 0.14
LT (mm)	0.02 ± 0.04	0.000	− 0.05 to 0.10
Kf (D)	0.22 ± 0.19	0.000	− 0.14 to 0.59
Ks (D)	0.04 ± 0.18	0.012	− 0.31 to 0.40
Km (D)	0.13 ± 0.13	0.000	− 0.13 to 0.40
AST (D)	− 0.18 ± 0.25	0.000	− 0.68 to 0.32
J_0_ (D)	0.12 ± 0.11	0.000	− 0.09 to 0.34
J_45_ (D)	0.09 ± 0.08	0.000	− 0.07 to 0.25
WTW (mm)	0.68 ± 0.40	0.000	− 0.11 to 1.47
PD (mm)	0.67 ± 0.84	0.000	− 0.97 to 2.31

*AL* = axial length; *CCT* = central corneal thickness; *AQD* = aqueous humor depth; *ACD *= anterior chamber depth; *LT* = lens thickness; *Kf* = flattest meridian keratometry; *Ks* = steepest meridian keratometry; *Km* = mean keratometry; *AST* = corneal astigmatism; *J*_*0*_ = astigmatism parameters J_0_; *J*_*45*_ = astigmatism parameters J_45_; *WTW* = white to white; *PD* = pupil diameter; *SD* = standard deviation; *LoA* = limits of agreement

## Discussion

Precise ocular biometry is crucial to the advancement of ophthalmology, necessitating ongoing updates to biometric instruments. Given the rapid development of new biometric technologies, it is essential to assess whether their measurements can be integrated into clinical practice and to validate their accuracy and precision. Reliability in clinical practice can only be assured through rigorous validation and comparison of data from emerging biometric devices, thereby enabling more precise diagnoses and enhanced treatment efficacy for patients [22]. The objective of this study is to assess the repeatability and reproducibility of the Colombo IOL instrument based on SD-OCT principle and their agreement with the IOLMaster 700 based on SS-OCT principle in normal subjects.

Our data demonstrated the high repeatability of the Colombo IOL in healthy young eyes. The S_w_ value of the AL in this study was 0.01 mm, and the ICC value was 1.000, showing excellent repeatability. In addition, AL also showed excellent reproducibility with an ICC value of 1.000. These results are similar to those previously reported in normal subjects with optical biometers based on a similar technology. Sikorski et al. used the Revo-NX (Optopol, Poland), based on the principle of SD-OCT, to measure AL in IOL eyes with repeatability and reproducibility ICC values of 1.000 [23]. Ni et al. using the AOCT-1000M (Aoying, China) based on the same SD-OCT principle as the Colombo IOL obtained an ICC value of 1.000 for AL [24]. Moreover, Domínguez-Vicent et al. analyzed a fully automated SS-OCT biometer, Eyestar 900 (Haag Streit AG, Koeniz, Switzerland), and obtained a low S_w_ value of 0.008 mm for AL [25].

SD-OCT has high resolution and faster image acquisition capability. The ICC, S_w_, TRT, and CoV of CCT measured by Rao et al. using RTVue were 0.990, 2.2 μm, 4.2 μm, and 0.4%, respectively [26]. Mansoori et al. used RTVue to measure CCT in normal subjects with an ICC of 0.994 [27]. The ICC values of CCT measured by Ni et al. using AOCT-1000 M and RTVue were 0.998 and 0.994, respectively [24]. Hong et al. used RTVue to measure a TRT value of 4.7 μm for CCT in normal subjects [28]. In our study, we observed better repeatability and reproducibility of CCT measured by the Colombo IOL in contrast to previous studies, by low S_w_ (first observer: 1.63 μm, second observer: 1.18 μm), TRT (first observer: 4.52 μm, second observer: 3.26 μm) and CoV (first measurer: 0.31%, second measurer: 0.22%). Moreover, the ICCs for CCT (first observer: 0.997, second observer: 0.998) were almost close to 1.000. The CCT measurements also showed excellent reproducibility with low S_w_ of 0.90 μm, TRT of 2.49 μm, and CoV of 0.17%.

Previous studies have shown that biometers based on FD-OCT have good repeatability in measuring AQD, ACD, and LT. Shetty et al. used IOLMaster 700 and Anterion to measure the repeatability of ACD in cataract patients with ICC of 0.9972 and 0.9999, respectively [29]. Fişuş et al. used the IOLMaster 700 and Anterion to measure the ACD of cataract patients with S_w_ < 0.135 mm and CoV < 0.373 mm. S_w_ of AQD was < 0.133 mm and CoV was < 0.366 mm [30]. Montés-Micó et al. used instruments such as the IOLMaster 700 and Lenstar LS 900 to measure LT values of normal human eyes with good repeatability and reproducibility [31]. Venkataraman et al. used the MS-39 (CSO, Italy) based on SD-OCT in combination with Placido disc principle and Eyestar 900 (Haag Streit AG, Koeniz, Italy) based on SS-OCT principle. The CoV values of ACD in healthy subjects measured were all < 1.2% [32]. In our study, the repeatability S_w_ and TRT values of AQD, ACD and LT were all < 0.04 mm and < 0.12 mm, respectively, the ICCs were all > 0.980. The reproducibility S_w_, TRT, ICCs of AQD and ACD were 0.02 mm, 0.06 mm and 0.995, respectively. The reproducibility of LT was good with S_w_ < 0.03 mm, CoV < 0.72% and ICC > 0.994. This demonstrates the excellent repeatability and reproducibility of AQD, ACD, and LT measurements with the Colombo IOL.

The Colombo IOL, utilizing radial scanning technology of SD-OCT, provided reliable corneal curvature data. This study found that the CoV for repeatability and reproducibility of Km measurements using the Colombo IOL was below 0.25%, and the ICC exceeded 0.994, demonstrating excellent repeatability and reproducibility. It would be beneficial to include comparisons with other device reports to highlight this performance. In our study, the AST parameters J_0_ and J_45_ measured by the Colombo IOL were found to be reliable. These results surpass other devices, such as the Cassini Color LED Corneal Analyzer (TRT: J_0_ = 0.42 D, J_45_ = 0.25 D), Humphrey Atlas 9000 based on Placido disc (TRT: J_0_ = 0.25 D, J_45_ = 0.39 D), and more so than the IOLMaster 700 based on SS-OCT (TRT: J_0_ = 0.33 D, J_45_ = 0.35 D) [33, 34].

### Comparisons between the Colombo IOL and IOLMaster 700 measurements

In healthy eyes, the present study found similar measurement results for AL using the Colombo IOL and IOLMaster 700. The MD was − 0.03 ± 0.03 mm, and the absolute value of the 95% LoA was 0.08 mm. Although the difference was statistically significant, it is negligible in clinical practice and can be considered high agreement [35].

In the measurement of CCT, the MD between them was − 13.34 ± 4.21 μm, and the maximum absolute value of 95% LoA was 21.58 μm. Ruan et al. used the CASIA 2 and IOLMaster 700 to measure the 95% LoA of CCT in cataract patients, ranging from − 30.06 to 0.43 μm [36]. The differences between the two instruments are clinically acceptable, and these differences are smaller in our study. This means that even if there are small differences in the measurement of CCT between the two instruments, these differences are acceptable for clinical diagnosis and treatment due to their low degree of correlation with intraocular pressure.

Lender et al. used the Eyestar 900 and IOLMaster 700 to measure the ACD measurements of patients before cataract surgery, and the difference was not statistically significant [37]. In our results, ACD values measured by the Colombo IOL and IOLMaster 700 were similar, with 95% LoA ranging from − 0.01 to 0.14 mm, and 95% LoA of AQD values ranging from 0.01 to 0.15 mm. Since each 0.10 mm deviation of the ACD only leads to an IOL refractive error of about 0.14 D [35], the narrow 95% LoA shows a high degree of agreement between the two instruments. On the other hand, the difference in LT was 0.02 ± 0.04 mm, with a 95% LoA of − 0.05 to 0.10 mm, indicating good agreement.

Consistency of Km measurement: Eibschitz-Tsimhoni et al. found that when K changes by 1.0 D, the IOL power calculation changes by 0.8–1.3 D [38]. Jasvinder et al. stated that the difference between 1.0 D and 0.5 D in K translates into a IOL power difference of around 1.0 D and 0.5 D [39]. To ensure proper visual acuity following IOL implantation, we set a clinical difference threshold at 0.5 D. Güçlü et al. used Pentacam and SS-OCT equipment to measure the corneal curvature of healthy subjects and keratoconus patients, and found that the measured values of the two were very close, and the difference was not statistically significant [40]. In our study, the 95% LoA of Km ranged from − 0.13 to 0.40 D and the absolute value of the 95% LoA (0.4 D) would still be below the threshold for clinical difference. Clearly, the two devices are clinically interchangeable when measuring Km.

Özyol et al. compared the Pentacam and IOLMaster 700 while measuring the 95% LoA of J_0_ in normal population from − 0.10 to 0.24 D, and the 95% LoA of J_45_ from − 0.31 to 0.27 D; they noted that the two devices had good consistency and could be used interchangeably [41]. The results of AST parameters measured by the Colombo IOL and IOLMaster 700 were found to be similar to those obtained previously. The mean J_0_ difference was 0.12 ± 0.11 D, and the maximum absolute value of 95% LoA was 0.34 D. The MD of J_45_ was 0.09 ± 0.08 D, and the maximum absolute value of 95% LoA was 0.25 D. These data showed the good agreement between these two biometers in measuring corneal AST.

Yang et al. used the IOLMaster 500, IOLMaster 700, and Argos devices to measure WTW in patients prior to cataract surgery [42]. The 95% LoA of WTW measured by the IOLMaster 500 and IOLMaster 700 was − 0.761 to 0.432 mm, and the 95% LoA of WTW measured by the IOLMaster 500 and Argos was − 1.641 to 0.631 mm. The 95% LoA measured by the IOL Master 700 and Argos ranged from − 1.458 to 0.748 mm. The IOLMaster devices measure the diameter of the corneal contour based on the camera image, while the Argos makes the measurement by identifying the junction between the cornea and the iris from OCT images. This different measurement method may have contributed to the differences in measurement results. Yang et al. also noted that when using the Holladay formula for calculating IOL power with WTW, the results of the two SS-OCT devices would be different [43]. In our study, the MD in WTW was 0.68 ± 0.40 mm, with a wide 95% LoA ranging from − 0.11 to 1.47 mm. Therefore, compared with the IOLMaster 700, the WTW measurements of the Colombo IOL tend to be larger because the Colombo IOL recognizes the junction between the cornea and the iris from OCT images, and these two instruments need to be used with caution when measuring WTW. When performing pre-cataract measurements and calculations, careful consideration should be given to the equipment used and the formula employed to ensure the accuracy of the diopter calculation.

Pupil size plays a crucial role in improving visual performance of biological and environmental factors, and age, illumination, and refractive error are important factors that affect pupil size [44]. In this study, the MD in PD was 0.67 ± 0.84 mm with a 95% LoA ranging from − 0.97 to 2.31 mm. The Colombo IOL tends to have larger PD measurements compared to the IOLMaster 700, and the two instruments need to be carefully interchanged in terms of PD measurements.

The detection of proportional bias for AL, CCT, and WTW suggests that as the magnitude of these parameters increases or decreases, the difference between the two devices also changes systematically. This highlights the need for caution when using these devices interchangeably for these particular measurements, especially in cases where extreme values of AL, CCT, or WTW are observed.

The limitations of this study include the absence of patients with ocular diseases such as cataract, glaucoma, and corneal diseases such as keratoconus and only enrolled young healthy myopic populations. Therefore, we need to further explore the repeatability, reproducibility, and consistency of the instrument measurements in disease states. Future studies should aim to broaden these findings to provide a more comprehensive comparison and evaluation of the performance differences among various instruments and their accuracy in measuring ocular biological parameters.

## Conclusions

Our findings provide valuable information for understanding the feasibility and consistency of the Colombo IOL in measuring normal human eyes. The new Colombo IOL shows excellent repeatability and reproducibility. In addition, the Colombo IOL and IOLMaster 700 have high agreement in measuring AL, CCT, AQD, ACD, LT, Kf, Ks, Km, AST, J_0_, and J_45_ parameters. In clinical practice, these two devices can be used interchangeably. However, the Colombo IOL may overestimate WTW and PD values compared to the IOLMaster 700, and these two instruments need to be used with caution for WTW and PD measurements.

## Data Availability

All data generated or analyzed during this study are included in this published article.

## Abbreviations

OCT: Optical coherence tomography
TD-OCT: Time-domain optical coherence tomography
FD-OCT: Fourier-domain optical coherence tomography
SD-OCT: Spectral-domain optical coherence tomography
SS-OCT: Swept-source optical coherence tomography
AL: Axial length
CCT: Central corneal thickness
ACD: Anterior chamber depth
AQD: Aqueous depth
K: Keratometry
AST: Astigmatism
WTW: White-to-white
PD: Pupil diameter
Kf: Flattest meridian keratometry
Ks: Steepest meridian keratometry
Km: Mean keratometry
S_w_: Within-subject standard deviation
TRT: Test–retest repeatability
CoV: Coefficient of variation
ICC: Intraclass correlation coefficient
MD: Mean difference
